# A homozygous missense variant in *VWA2*, encoding an interactor of the Fraser-complex, in a patient with vesicoureteral reflux

**DOI:** 10.1371/journal.pone.0191224

**Published:** 2018-01-19

**Authors:** Amelie T. van der Ven, Birgit Kobbe, Stefan Kohl, Shirlee Shril, Hans-Martin Pogoda, Thomas Imhof, Hadas Ityel, Asaf Vivante, Jing Chen, Daw-Yang Hwang, Dervla M. Connaughton, Nina Mann, Eugen Widmeier, Mary Taglienti, Johanna Magdalena Schmidt, Makiko Nakayama, Prabha Senguttuvan, Selvin Kumar, Velibor Tasic, Elijah O. Kehinde, Shrikant M. Mane, Richard P. Lifton, Neveen Soliman, Weining Lu, Stuart B. Bauer, Matthias Hammerschmidt, Raimund Wagener, Friedhelm Hildebrandt

**Affiliations:** 1 Division of Nephrology, Department of Medicine, Boston Children’s Hospital, Harvard Medical School, Boston, Massachusetts, United States of America; 2 Center for Biochemistry, Center for Molecular Medicine Cologne, University of Cologne, Cologne, Germany; 3 Department of Pediatrics, Cologne Children’s Hospital, Cologne, Germany; 4 Institute of Zoology-Developmental Biology, Biocenter Cologne, Center for Molecular Medicine Cologne, University of Cologne, Cologne, Germany; 5 Institute for Dental Research and Oral Musculoskeletal Biology, Medical Faculty, University of Cologne, Cologne, Germany; 6 Talpiot Medical Leadership Program, Sheba Medical Center, Tel-Hashomer, Israel; 7 Department of Nephrology, Children’s Hospital of Fudan University, Shanghai, China; 8 Division of Nephrology, Department of Medicine, Kaohsiung Medical University, Kaohsiung, Taiwan; 9 Department of Pediatric Nephrology, Dr. Mehta’s Multispeciality Hospital, Chennai, India; 10 Department of Pediatric Nephrology, Institute of Child Health and Hospital for Children, the Tamil Nadu Dr. M.G.R. Medical University, Chennai, Tamil Nadu, India; 11 Medical Faculty Skopje, University Children’s Hospital, Skopje, Macedonia; 12 Division of Urology, Department of Surgery, Nazarbayev University, Astana, Kazakhstan; 13 Department of Genetics, Yale University School of Medicine, New Haven, Connecticut, United States of America; 14 Howard Hughes Medical Institute, Chevy Chase, Maryland, United States of America; 15 Department of Pediatrics, Center of Pediatric Nephrology & Transplantation, Cairo University, Egyptian Group for Orphan Renal Diseases, Cairo, Egypt; 16 Renal Section, Department of Medicine, Boston University Medical Center, Boston, Massachusetts, United States of America; 17 Department of Urology, Boston Children’s Hospital and Harvard Medical School, Boston, Massachusetts, United States of America; National Cancer Institute, UNITED STATES

## Abstract

Congenital anomalies of the kidney and urinary tract (CAKUT) are the most common cause (40–50%) of chronic kidney disease (CKD) in children. About 40 monogenic causes of CAKUT have so far been discovered. To date less than 20% of CAKUT cases can be explained by mutations in these 40 genes. To identify additional monogenic causes of CAKUT, we performed whole exome sequencing (WES) and homozygosity mapping (HM) in a patient with CAKUT from Indian origin and consanguineous descent. We identified a homozygous missense mutation (c.1336C>T, p.Arg446Cys) in the gene *Von Willebrand factor A domain containing 2* (*VWA2*). With immunohistochemistry studies on kidneys of newborn (P1) mice, we show that Vwa2 and Fraser extracellular matrix complex subunit 1 (Fras1) co-localize in the nephrogenic zone of the renal cortex. We identified a pronounced expression of Vwa2 in the basement membrane of the ureteric bud (UB) and derivatives of the metanephric mesenchyme (MM). By applying *in vitro* assays, we demonstrate that the Arg446Cys mutation decreases translocation of monomeric VWA2 protein and increases translocation of aggregated VWA2 protein into the extracellular space. This is potentially due to the additional, unpaired cysteine residue in the mutated protein that is used for intermolecular disulfide bond formation. VWA2 is a known, direct interactor of FRAS1 of the Fraser-Complex (FC). FC-encoding genes and interacting proteins have previously been implicated in the pathogenesis of syndromic and/or isolated CAKUT phenotypes in humans. *VWA2* therefore constitutes a very strong candidate in the search for novel CAKUT-causing genes. Our results from *in vitro* experiments indicate a dose-dependent neomorphic effect of the Arg446Cys homozygous mutation in *VWA2*.

## Introduction

Congenital anomalies of the kidney and urinary tract (CAKUT) constitute the most common cause of chronic kidney disease (CKD) in the first three decades of life (~ 50%) and are very frequently encountered birth defects (3-6/1,000 live births) [1–4]. CAKUT comprises a wide phenotypic spectrum that ranges from renal agenesis to vesicoureteral reflux (VUR) [1,5]. The pathogenesis of CAKUT is very heterogeneous and remains to further be elucidated. However, a disturbed embryonic development of tissues derived from the ureteric bud (UB) and/or the metanephric mesenchyme (MM) appears to be a shared feature of a majority of CAKUT manifestations [5].

Several lines of evidence indicate that CAKUT may be caused by mutations in single monogenic genes by point mutations [3,5,6] as well as copy-number variations (CNV) [7,8]. Supporting evidence includes, i) the presence of CAKUT phenotypes in known monogenic multi-organ human syndromes, ii) familial occurrence of CAKUT, iii) the presence of monogenic mouse models with CAKUT as well as iv) the fact that the development of the kidney and urinary tract is governed by distinct developmental genes [2,3,7,9]. To date approx. 40 monogenic causes for CAKUT have been identified [10]. However, only ~20% of cases with CAKUT can be explained by mutations in those genes [6]. The identification of CAKUT genes is often complicated by incomplete penetrance and variable expressivity [5,10]. Additionally, the very broad locus heterogeneity makes it challenging to identify multiple individuals with disease-causing mutations in the same gene even when studying large cohorts [10–12].

Recessive *missense mutations* in genes encoding members of the Fraser complex (FC) or associated proteins were recently identified in cases of isolated, human CAKUT [1].. In contrast, *truncating* mutations in these genes result in lack of Fraser complex proteins and constitute an established genetic cause of Fraser syndrome (FS). FS is a syndromic disorder that is characterized by defects in kidney development as well as embryonic epidermal blistering, cyptophthalmos and syndactyly [13]. The identification of new molecular causes of CAKUT helps improve the knowledge about physiological processes underlying the embryonic development of the kidney and urinary tract. In fact, as an increasing number of diverse single-gene causes of CAKUT are identified, they have started to coalesce around distinct molecular pathways [3,6,14–16]. Interactors of previously identified CAKUT genes/proteins consequently constitute promising candidates in the search for novel pathogenic causes. VWA2 (AMACO) [17] represents such a candidate. It was recently identified as a novel member of the FC by its interaction with the CSPG domains of Fras1. Matrix deposition of VWA2 is lost in Fras1 deficient mice and zebrafish [18].

We here present a patient from consanguineous descent and Indian origin with high-grade, bilateral VUR resulting in end-stage renal disease (ESRD), the most advanced form of CKD, requiring renal-replacement therapy for patient survival, in whom we performed whole exome sequencing (WES). We discovered a homozygous missense mutation (Arg446Cys) in the gene *Von Willebrand factor A domain containing 2* (*VWA2*).

## Materials and methods

### Ethics statement

We obtained blood samples, pedigrees, and clinical information after receiving written, informed consent (http://www.renalgenes.org) from all patients. Approval for experiments on human DNA was obtained from the Boston Children’s Hospital Internal Review Board (IRB) and the review board of the University of Michigan (protocol # IRB-P00006200). Mouse experiments were approved by the Boston Children’s Hospital (BCH) Institutional Animal Care and Use Committee (IACUC). All national and institutional guidelines for the care and use of laboratory animals were followed. Euthanasia was performed according to local protocols. In detail, sacrifice of neonatal mice was performed by decapitation with scissors. All efforts were made to minimize suffering.

### Homozygosity mapping

For genome-wide homozygosity mapping, the GeneChip Human Mapping 250K *StyI* Array from Affymetrix was used. Nonparametric LOD scores were calculated using a modified version of the program GENEHUNTER 2 through stepwise use of a sliding window with sets of 110 SNPs [19,20]. The program ALLEGRO was employed to identify regions of homozygosity by descent as described using a disease allele frequency of 0.0001 and CEU marker allele frequencies [21].

### Whole exome capture and next generation sequencing

Genomic DNA (2 μg) from the affected individual was fractured and exome capture was performed with a customized Agilent SureSelect All Exome Kit v2.0 (Agilent Technologies, Santa Clara, CA, USA), according to manufacturer's protocol. The library was sequenced on an Illumina MiSeq^™^ sequencing platform. Image analysis and base calling were generated by the Illumina pipeline using default parameters.

### Sequence alignment, and variant calling

All sequence alignments to the human genome reference genome (hg19) and variant calling were performed using the CLC Genomics Workbench^™^ (version 6.5.1) software (CLC bio, Aarhus, Denmark) software. Only variants with an allelic ratio between alternative and reference of 0.8 of higher were called as potential homozygous variants. Variants that did not meet these criteria were consequentially interpreted as heterozygous variants. Trimmed sequence reads were mapped to the human reference genome (hg19) using the Map Reads to Reference program with the following settings: mismatch cost = 2, insertion cost = 3, deletion cost = 3, length fraction = 0.5, similarity fraction = 0.9 and map to nonspecific reads = “randomly”. The non-specific reads were then ignored for count and coverage. All variants with a minimum coverage of 2 were used. The variations in the samples were called using probabilistic variant detection using CLC. All the called variants were then annotated and evaluated.

### Variant annotation and evaluation

All called variants were annotated using CLC Genomics^™^ Pre-built programs. Variation annotation was performed using the “Amino Acid Changes”, “Annotate with Overlap Information”, and “Annotate from Known Variants” programs. The variants were annotated with the conservation scores (phastCons and PhyloP), 1,000 Genomes database, dbSNP database, ExAC, EVS, SNP138. As a first step, the variants were filtered out for SNP138 common (Minor Allele Frequency > 1%). All synonymous variants and intronic variants that were not located within a splice site region were then excluded. The rest of the variants were considered for further evaluation. Remaining variants were then ranked based on their probable impact on the function of the encoded protein considering evolutionary conservation among orthologues across phylogeny, as well as web-based prediction programs (PolyPhen-2 [22], SIFT [23], and Mutation Taster [24]). The variants were ranked based on their effect as described above. Allele Frequency of >1% in the EVS or ExAC database and non-segregation were also considered as exclusion criteria. The identified mutations were confirmed by Sanger sequencing of genomic DNA.

### High throughput *VWA2* variant analysis

Target sequencing of all coding exons and adjacent splice sites of *VWA2* was performed in 864 additional individuals from 589 families with CAKUT. The patients were derived from different pediatric nephrology centers worldwide. Exons were amplified using microfluidic technology (Fluidigm) and subsequently sequenced using a next-generation sequencing technique as described previously [3,25]. PCR primer sequences are available upon request. Variants were confirmed by Sanger sequencing, and tested for segregation with the respective CAKUT phenotype.

### Generation of antibodies against human VWA2, murine VWA2 and murine nephronectin

A cDNA construct coding for murine nephronectin was generated by RT-PCR using forward primer 5’ aaaTCTAGAgacttcgacgggaggtggcccaa 3’ and reverse primer 5’ tattGCGGCCGCtcagcagcgacctcttttcaagct 3’, cloned with 5′-terminal *XbaI* or 3′-terminal *NotI* restriction sites and inserted into a modified pCEP-Pu vector containing an N-terminal BM-40 signal peptide [26] followed by an N-terminal One-STrEP-tag (IBA GmbH, Göttingen, Germany) upstream of the restriction sites. The recombinant expression and affinity purification of murine nephronectin was performed as described below for other recombinant proteins. The affinity purified nephronectin and the recombinantly expressed P3 fragments of human and murine VWA2 [27] were used for immunization. Specific antibodies were purified by affinity chromatography on columns with antigen coupled to CNBr-activated Sepharose (GE Healthcare). The specific antibodies were eluted with 0.1 M glycine, pH 2.5, the eluate neutralized with 1 M Tris–HCl, pH 8.8, and adjusted to 150 mM NaCl.

### cDNA cloning, site directed mutagenesis and expression of Arg446Cys VWA2

A full-length human VWA2 expression construct was generated by PCR on a full-length human AMACO cDNA clone in pBKS to introduce 5’-terminal *NheI* and 3’-terminal *NotI* cloning sites using the forward primer 5’-CAATGCTAGCctccaggaagtccatgtaagc-3’ and the reverse primer 5’-CAATGCGGCCGCtcaggggcgtctcaagaatc-3’. The amplified PCR product was inserted into a modified pCEP-Pu vector containing an N-terminal BM-40 signal peptide [26], followed by an N-terminal One-STrEP-tag (IBA GmbH, Göttingen, Germany) upstream of the restriction sites. For site directed mutagenesis the full length VWA2 expression construct harboring the introduced cloning sites was cloned into the pBKS vector. Q5 High-Fidelity DNA Polymerase (NEB) using the forward primer 5’- GACCGGCCATGTAGAGTGGTG-3´ and the reverse primer 5’- CACCACTCTACATGGCCGGTC-3’ introduced the Arg446Cys mutation in both strands and after digestion of the methylated, nonmutated parental DNA template with DpnI, the mutated DNA was transformed into DH5α competent cells. A successfully mutated clone was recloned into the pCEP-Pu vector. The wild type and mutated expression constructs were transfected into 293 EBNA (Epstein–Barr virus nuclear antigen) cells with FuGENE HD (Roche, Mannheim, Germany) according to the manufacturer’s instructions. The cells were cultured 2 days in the presence of 10% fetal calf serum before harvest of cells and cell culture supernatant.

### Protein extraction and purification of Arg446Cys VWA2

For protein extraction two days after transfection, wild type and mutant VWA2 expressing cells were washed once with PBS and then scraped from the culture dish with 1 × SDS sample buffer (2% (w/v) SDS, 10% (v/v) glycerol, 0.05% (w/v) bromphenol blue, and 62,5 mM Tris-HCl, pH 6.8), mixed vigorously and put on ice for 30 minutes. The lysates were boiled for 5 minutes and centrifuged for 5 minutes. Cell culture supernatants were centrifuged for 10 minutes. For expression of larger amounts of wild type and mutant VWA2, cells were grown on 9 cm cell culture dishes and selected with puromycin (1 μg/ml). 100 ml medium were collected and wild type and mutant recombinant VWA2 were purified directly from serum-containing cell culture medium. After filtration and centrifugation (1 h, 10,000 × g), the cell culture supernatants were applied to a Streptactin column (0.2 ml, IBA GmbH) and eluted with 2.5 mM desthiobiotin, 10 mM Tris–HCl, pH 8.0.

### SDS-PAGE, agarose-polyacrylamide composite gel electrophoresis and immunoblot analysis of Arg446Cys VWA2

Proteins were analyzed on 4–12% (w/v) SDS-polyacrylamide gels under reducing and nonreducing conditions and on agarose-polyacrylamide (0.5% / 2.4%) composite gels under non-reducing conditions with samples treated with 2 M urea. After blotting to nitrocellulose recombinant VWA2 was detected by using polyclonal antibodies against the One-STrEP-tag or against human VWA2 diluted in TBS/5% milk powder. Bound antibodies were detected by luminescence using a peroxidase-conjugated swine anti-rabbit secondary antibody (Dako, Hamburg, Germany).

### Immunohistochemistry

Immunohistochemistry was performed on paraffin-embedded sections of newborn (P1) mice. Mouse experiments were approved by the Boston Children’s Hospital (BCH) Institutional Animal Care and Use Committee (IACUC). All national and institutional guidelines for the care and use of laboratory animals were followed. Euthanasia was performed according to local protocols. In detail, sacrifice of neonatal mice was performed by decapitation with scissors. All efforts were made to minimize suffering. After rehydration, sections were incubated in TRIS-EDTA buffer (pH 9.0, Vector Laboratories) at 95°C for 20 mins for epitope retrieval. Following cooling, sections were permeabilized using 0.025% triton (BioRad) and subsequently blocked for 1h in PBS containing 10% Goat Serum (Sigma) and 1% bovine serum albumin (Sigma). Sections were incubated with the respective affinity-purified antibodies (guinea pig-mouse anti-Vwa2, 1:300; rabbit-mouse anti-Fras1 (raised against CSPG-domain) [18], 1:500; rabbit-mouse anti-Npnt, 1:400) overnight at 4°C, using the indicated dilutions. Primary antibodies were visualized by subsequent addition to the sections of a secondary goat anti-guinea pig antibody (488 nm) or goat-anti-rabbit antibody (594 nm) (both Thermo Scientific), respectively. All secondary antibodies were used at a 1:1,000 dilution (diluted in blocking buffer). Sections were imaged using a Leica SP5X system with an upright DM6000 microscope or a A1R confocal microscope (Nikon Instruments).

### RT-PCR analysis of XBP-1 splicing

Total RNA was isolated from non-transfected, ER stress induced (1mM DTT for 2h), wild type VWA2 transfected and Arg446Cys VWA2 transfected 293EBNA cells using the RNeasy kit (Qiagen). RNA (1μg) was reverse transcribed with Superscript II (Thermo Fisher Scientific) and amplified with the Roche Expand High Fidelity PCR System using primers flanking the ER stress-responsive XBP-1 splice site. The primer sequences were: Forward 5’GGA GTT AAG ACA GCG TTG G3’ and reverse 5’ACT GGG TCC AAG TTG TCC AG3’. As a reference control an actin fragment was amplified using primers: Forward 5’CTT CTA CAA TGA GCT GCG TGT GG3’ and reverse 5’CTC ATT GCC AAT GGT GAT GAC C3’.

## Results

### Identification of the Arg446Cys mutation in individual A3964-21 with CAKUT

**Fig 1:**
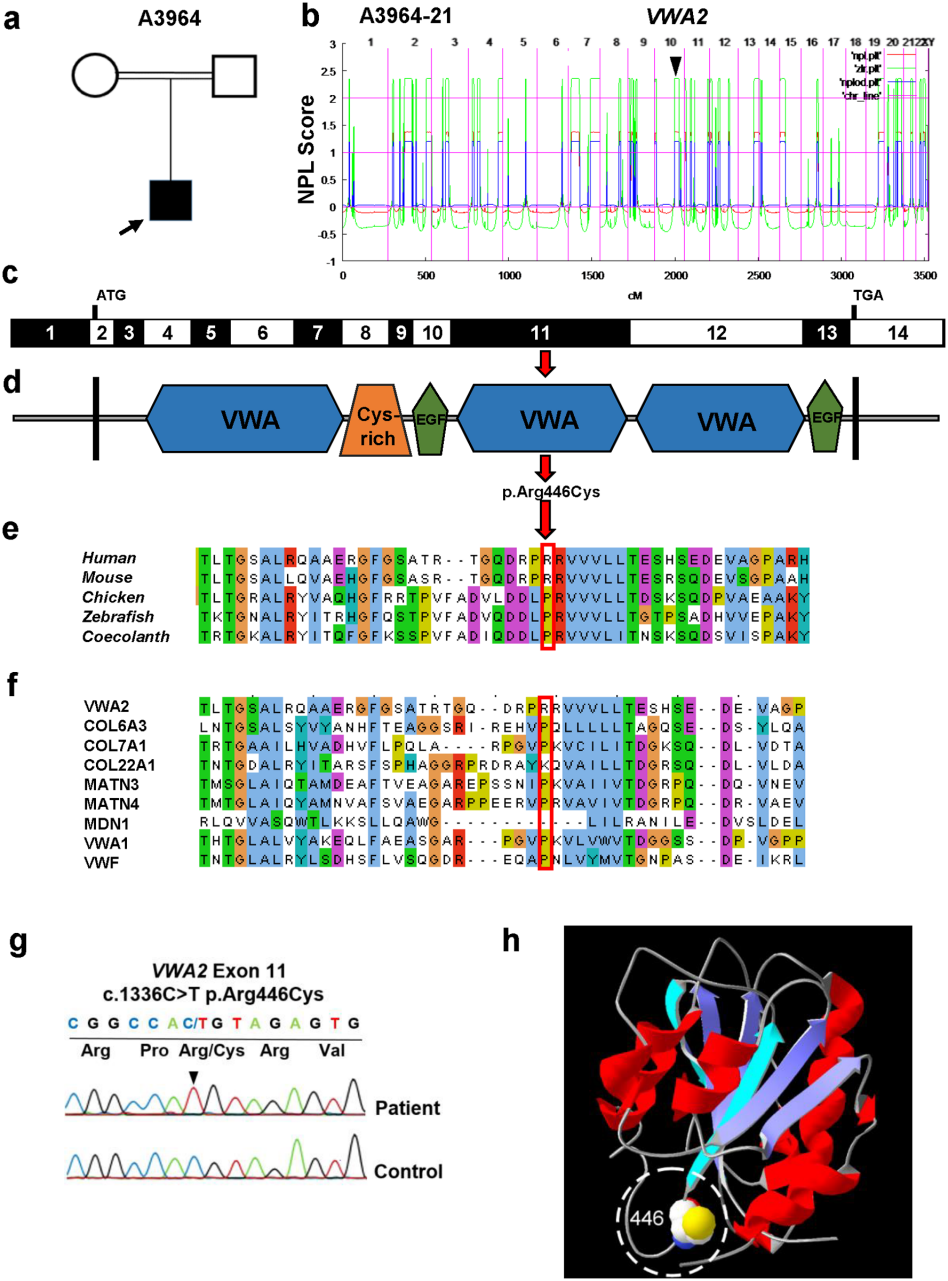
Homozygous variant identified in *VWA2* in a consanguineous individual with CAKUT. **(a)** Pedigree of consanguineous family (A3964) with affected individual A3964-21 (arrow). Squares represent males, circles represent females. Filled symbol indicates affected individual A3964-21. **(b)** Homozygosity mapping identifies recessive candidate loci in individual A3964-21. Non-parametric LOD scores (NPL) were calculated and plotted across the human genome. The X-axis shows single-nucleotide polymorphism positions on human chromosomes concatenated from p-ter (left) to q-ter (right). Genetic distance is given in cM. The *VWA2* locus (arrowhead) is positioned within a maximum NPL peak on chromosome 10. **(c)** Exon structure of human *VWA2* cDNA. **(d)** Protein domain structure of VWA2. **(e)** Evolutionary amino acid sequence conservation of VWA2. Arginine in position 446 is outlined in red. **(f)** Alignment of selected proteins with regions similar to second VWA-domain of VWA2. Arginine in position 446 is outlined in red. **(g)** Chromatograms obtained by direct sequencing of PCR products reveal homozygous variant c.1366C>T in exon 11 of the *VWA2* gene in individual A3964-21 compared to DNA from a healthy control (indicated by arrowhead). Parental samples were not available for segregation analysis. **(h)** 3D model of the second VWA domain of VWA2 (Glu339-Cys528) reveals that the mutant cysteine residue in position 446 (encircled by white dashed line) is partially surface exposed. The model was created using the protein structure homology-modelling server SWISS MODEL [34].

We studied a 12 year-old male (A3964-21) from India, who, at the age of 12 was diagnosed with bilateral VUR grade 5 and ESRD, the most advanced form of CKD. As his parents were consanguineous and unaffected, we hypothesized that the cause of CAKUT in this patient was due to a recessively inherited mutation (Fig 1a). While appearing the likeliest scenario, the possibility of a dominant inheritance of a variant with incomplete penetrance cannot be excluded in this setting.

Homozygosity mapping (HM) yielded 35 segments of homozygosity with a total cumulative genomic length of 440 Mb (Fig 1b). Following WES, 5 potentially disease causing homozygous variants were detected within the homozygous peak regions and confirmed with Sanger sequencing. No homozygous truncating variant was identified. The missense mutations were detected within the following 5 genes: *Von Willebrand Factor A Domain Containing 2*, *VWA2* (c.1336C>T, p.Arg446Cys, NM_001272046.1); *Tubulin Tyrosine Ligase Like 12*, *TTLL12* (c.1873G>A, p.Asp625Asn, NM_015140.3); *Testis Specific Serine Kinase 4*, *TSSK4* (c.104C>T, p.Ser35Phe, NM_001184739.1); *Neural EGFL Like 2*, *NELL2* (c.1841T>C, p.Val563Ala, NM_001145107.1) and *ATPase Phospholipid Transporting 10D (Putative)*, *ATP10D* (c.1645G>A, p.Val549Met, NM_020453.3). All 5 missense variants yielded predominantly deleterious prediction scores (≥ 3 out of 4 deleterious scores) in Poly Phen 2 (http://genetics.bwh.harvard.edu/pph2/), Mutation Taster (http://www.mutationtaster.org/), Sorting Intolerant From Tolerant (SIFT, http://sift.jcvi.org) and Combined Annotation Dependent Depletion (CADD, http://cadd.gs.washington.edu), and occur rarely in the general population (all identified variants MAF ≤ 0. 075% in ExAC and ≤ 0.06% in Gnomad). All identified variants demonstrate a higher MAF in the in the Indian (South-Asian) population, they, however, still remain rare (MAF of all variants in the South-Asian subpopulation ≤ 0. 48% in ExAC and ≤0.4% in GnomAD).

We then evaluated all variants in regards to a possible contribution to renal morphogenesis in general and specifically the CAKUT phenotype of our patient. None of the identified 5 genes had been previously reported in connection with a defined phenotype in OMIM. Furthermore, only 1 gene (*Nell2*) was reported in the MGI database (Mouse genome informatics, http://www.informatics.jax.org) to date. The identified phenotype however, does not include malformations of the renal or urinary system (MGI ID: 97490). Data obtained by Gudmap (GenitoUrinary Molecular Anatomy Project, http://www.gudmap.org) revealed, that 2 (Atp10d and Nell2) out of the 5 identified genes/proteins do not reach significant expression levels in the murine kidney in cell types relevant to the pathogenesis of CAKUT (e.g. ureteric bud or metanephric mesenchyme). *ATP10D* and *NELL2* were consequently not considered highest priority within the list of identified genes in our patient. The remaining 3 genes/ proteins (Vwa2, Ttll12, Tssk4) demonstrate an expression in CAKUT-relevant cell types in the murine kidney (Gudmap). Tssk4, of note, hereby demonstrates a strong, rather unspecific, expression in various additional cell types including small blood vessels, podocytes, and interstitial cells.

A subsequent literature research revealed that *TSSK4* and *ATP10D* have not been implicated in pathways related to kidney development to date. *TSSK4* appears to have a rather specialized role for male fertility [28] while sequence variants in *ATP10D* have been demonstrated to significantly associate with metabolic disorders including insulin resistance [29]. *NELL2* and *TTLL12* however, remotely appear connected to pathways that have been previously discussed in the development of CAKUT. Nell2 has been implicated as a ligand for Robo3 during the development of the central nervous system [30]. Members from the ROBO family of proteins (most prominently ROBO2) have previously been demonstrated to play a role in the pathogenesis of CAKUT [15,31]. However, to date *ROBO3* has not been shown to haven an impact on the development of the urinary tract and mice deficient for either *Robo3* or *Nell2* do not exhibit CAKUT as part of their phenotypic spectrum. TTLL12 has been recently published as a negative regulator of retinoic-acid-inducible gene I (RIG-I)- signaling [32]. RIG-I signaling is utilized by virus-infected cells to recognize viral RNA and activate IFN expression. Retinoic acid (RA) itself as well as genes immediately involved in RA-metabolism and signaling can be causes of (predominantly murine) CAKUT [33].

Taking into consideration results from this literature review, the gene *VWA2* was, however, considered the strongest candidate gene and was therefore selected for further assessment in regards to a potential role to the development of CAKUT in our patient (please see Discussion section for details on supporting literature). *VWA2* was subsequently included in targeted exon sequencing in our in-house world-wide cohort of >1,500 patients with CAKUT for the identification of additional patients with comparable phenotypes and mutations in this gene (please see Method section for details). The genes *VWA2*, *TTLL12*, *NELL2*, and *TSSK4* were furthermore submitted to the online database Genematcher (https://genematcher.org). Genematcher facilitates the exchange of genetic and phenotypic information by connecting clinicians and researchers world-wide. However, none of the genes (*VWA2*, *TTLL12*, *NELL2* and *TSSK4*) yielded positive findings (if any) in regards to CAKUT (please see S1 Table for more detailed information on filtering of identified variants).

The homozygous missense mutation (c.1336C>T, p.Arg446Cys) in VWA2 was identified in exon 11 of the *VWA2* gene (NM_001272042.1) (Fig 1c–1f). The Arg446 residue is located within the second von Willebrand factor A domain (VWA) of the protein (Fig 1d). Fig 1e depicts the conservation of the Arg residue in position 446 across evolution. Although the Arg residue in position 446 is not well conserved, neither of the species listed in the Ensembl database (www.ensembl.org) carries the observed change (Arg446Cys) (data not shown). This is reflected in deleterious conservation scores in Polyphen 2 (0.95) (http://genetics.bwh.harvard.edu/pph2/), (Table 1) phastCons (0.79) (http://compgen.cshl.edu/phast/) and phyloP (4.32) (http://compgen.cshl.edu/phast/help-pages/phyloP.txt) respectively. The mutation furthermore yielded the score “disease causing” (p-value 0.997) in the Mutation Taster software (http://www.mutationtaster.org/). None of the species listed in the Ensembl database has a cysteine residue in regions adjacent to position 446 (data not shown) which suggests a severe change in the physicochemical properties of this region by the introduction of a cysteine residue *via* the Arg446Cys mutation of individual A3964-21. This is further supported by a multiple sequence alignment of the second VWA2 domain of VWA2 (location of Arg446Cys) (Fig 1f) which reveals, that structurally related proteins with similar VWA-domains do not have a cysteine residue in the position corresponding to 446 of VWA2 as well as in the adjacent regions (except for one protein, COL7A1).

**Table 1:** Disease-causing mutations and phenotypes of individual A3964-21 with end-stage renal disease and homozygous missense mutation in *VWA2*.

Individual	Origin	Causative Gene	Genomic coordinates (hg19)	Alteration in coding sequence	Altera-tion of protein	Zygo-sity	Contin-uous AA sequence conservation	PP2 MutTast SIFT CADD	ExAC	Gnomad	Pheno-type
A3964-21	India	*VWA2*	chr10:116046036C>T	c.1336C>T	p.Arg446Cys	Homo	*Mus musculus*	P.D. (0.95); D.c. (0.997); Tol. (0.1); 29.0	0/54/119156 (MAF 0.046%)	2/114/274368 (MAF 0.041%)	bilat. VUR (V), ESRD

AA, amino acid; PP2, Poly Phen 2; MutTast, Mutation Taster; SIFT, Sorting intolerant from tolerant; CADD, Combined Annotation Dependent Depletion; Homo, homozygous; P.D., probably damaging; D.c., Disease causing; Tol, tolerated; VUR, vesicoureteral reflux; ESRD, end-stage renal disease.

The homozygous variant (c.1336C>T) was confirmed by exon-PCR and Sanger sequencing in the affected individual (Fig 1g). Parental DNA was not available for segregation analysis. Individual A3964-21 was additionally screened for variants in all 38 currently established monogenic causes for CAKUT, ~200 syndromic forms of CAKUT, and CAKUT candidate genes derived from ~180 mouse models for CAKUT, as well as CNVs that include loci of known CAKUT genes, without detecting any competing potentially causative mutations [2,3].

In order to further evaluate potential functional deleteriousness of the Arg446Cys variant, we modeled it on the structure (4cn9) of a VWA domain of PTMP1, a collagen-binding matrix protein that has 28% sequence identity and 42% sequence similarity with the second VWA domain of VWA2. Like all VWA domains this domain is composed of a series of alternating β-strands and α-helices forming a central parallel β-sheet that is surrounded by amphipathic α-helices. This structure is well conserved also in the second VWA domain of VWA2 and comprises the classical “Rossmann fold” of VWA domains, indicating that the model is reliable. The 3D modelling (Fig 1h) reveals the partially surface exposed location of residue Arginine 446 opposite to the MIDAS motif on top of the domain. It is located just in front of the fourth β-strand that contributes to the central hydrophobic β-sheet whose integrity is predicted to be unaffected by the mutation. Interestingly, the cysteine residue lays in the vicinity of the N- and C-terminal cysteine residues of the VWA domain and could allow formation of novel disulfide bonds and disulfide shuffling.

The identified change (c.1336C>T, p.Arg446Cys) is a known SNP (rs148731211). Its heterozygous state appears to occur rarely (54 times in ~60,000 individuals; minor allele frequency (MAF) 0.05%) in the overall population gathered in the Exome Aggregation Consortium database (ExAC) and, while it does show an enrichment in the south Asian subpopulation (38 times heterozygously in ~7,900 south-Asian individuals, MAF 0.241%), rs148731211 to date has not been reported homozygously in the population databases Exome Variant Server (EVS) and ExAC in which the Indian (South Asian) subpopulation is well represented. A screening for variant rs148731211 in an in-house Indian cohort comprising 50 individuals did not reveal any homozygous carriers. While being encouraging the limited sample size restricts futher conclusions regarding MAF and ascertainment of homozygous carriers.

### High-throughput variant analysis indicates that VWA2 mutations are a rare cause of CAKUT

Targeted sequencing of all 14 *VWA2* exons in 864 additional individuals with CAKUT did not reveal additional individuals with recessive mutations in this gene. This finding indicates, that *VWA2* most likely is a rare cause of CAKUT and is in line with the distinct genetic heterogeneity of CAKUT which has been described previously [10–12,35,36].

### Vwa2 partially co-localizes with Fras1 and Npnt in the nephrogenic zone of the murine renal cortex

**Fig 2:**
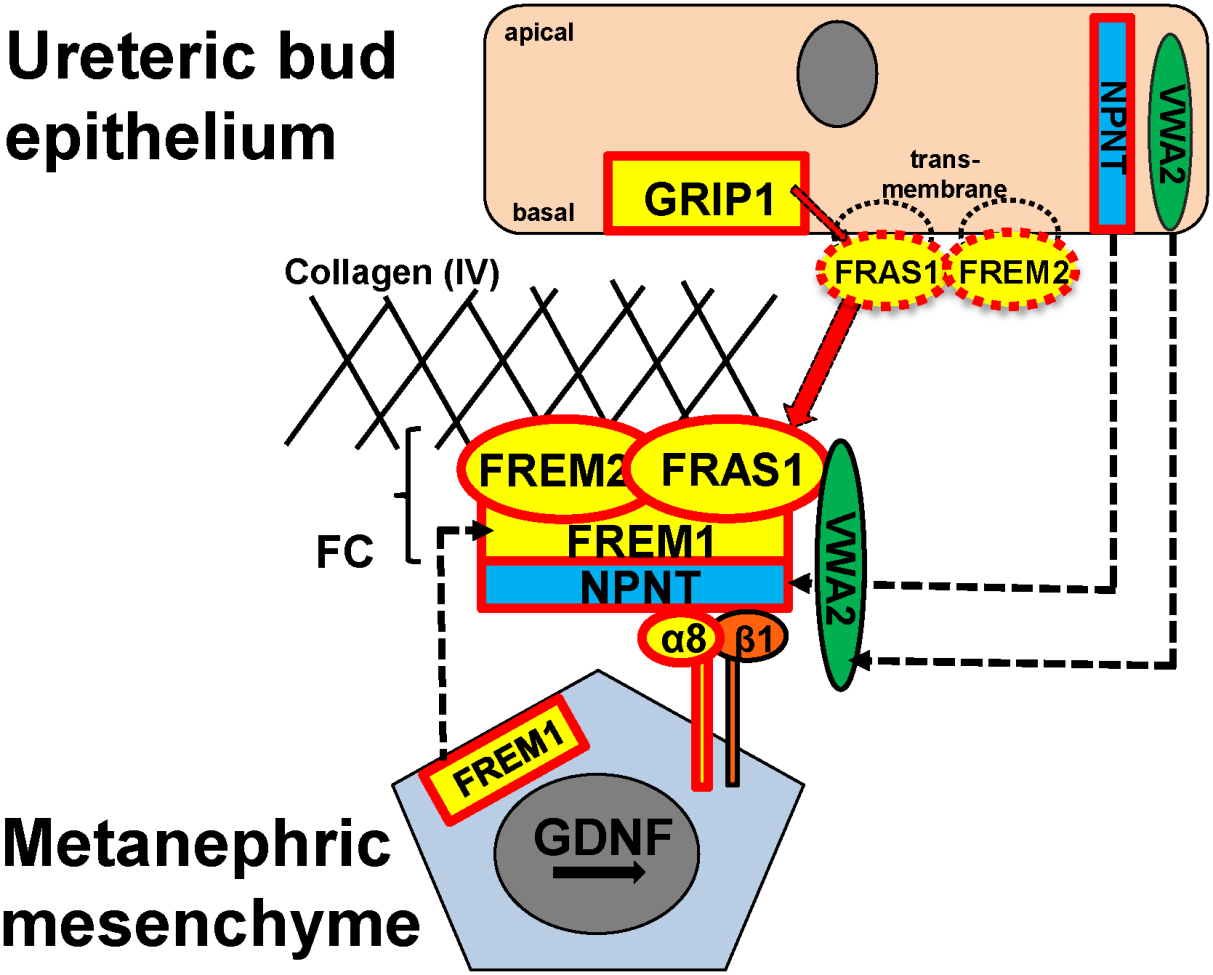
Mutations in genes encoding components and interactors of the basement-membrane associated Fraser-complex constitute known monogenic causes of isolated CAKUT.

**Fig 3:**
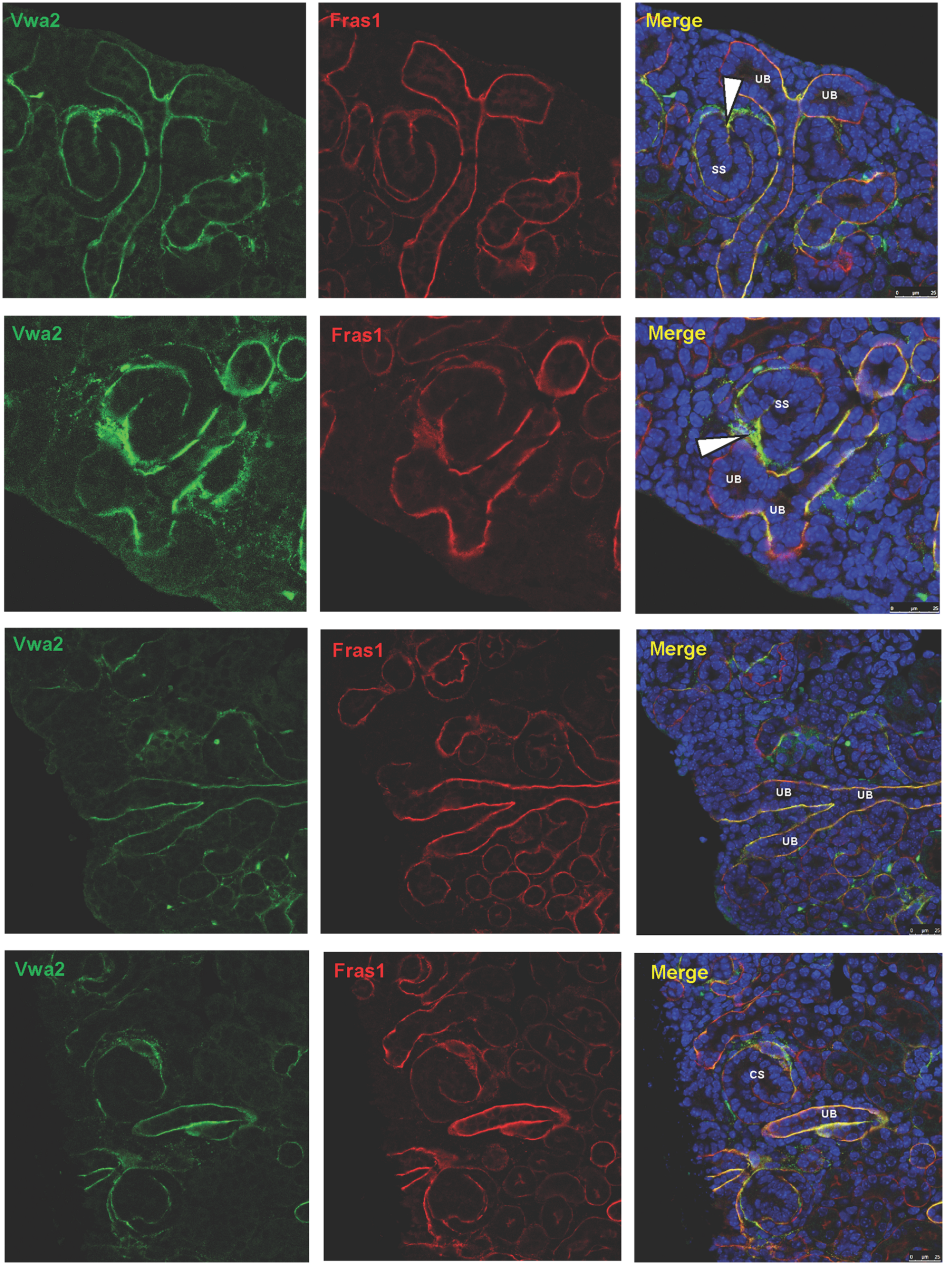
Vwa2 co-localizes with Fras1 in the nephrogenic zone of newborn mice. Immunohistochemistry on coronal sections of the nephrogenic zone of newborn (P1) mice. Figures display representative staining results obtained from kidney sections of two different animals. Experiments were performed independently and yielded similar results. Staining for Vwa2 (green) and Fras1 (red) demonstrates co-localization of the Vwa2 and Fras1 proteins in the in the renal cortex of newborn mice (ureteric bud (UB) as well as derivatives of the metanephric mesenchyme (MM), comma-shaped bodies (CS), and S-shaped bodies (SS)) as seen by the yellow-appearing structures in the “merge” panel on the very right. Vwa2 (green) hereby always co-localizes with Fras1 (red). Vwa2 expression (green) is particularly pronounced in the superior aspect of the S-shaped body, at the border between the developing distal tubule and the proximal tubule portion (indicated by arrowheads). Fras1 (red), however, appears to have a more abundant expression pattern and is partially expressed where Vwa2 is not present.

To evaluate a potential role of VWA2 in renal development, we performed immunohistochemistry studies on coronal sections of kidneys from newborn (P1) mice. Special attention was paid to the nephrogenic zone of the renal cortex. VWA2 is an interactor of Fras1 of the Fraser-complex (FC) [18] (Fig 2). Results from immunohistochemistry experiments demonstrated colocalization of Vwa2 with Fras1 in the nephrogenic zone of the newborn murine kidney (Fig 3). Vwa2 and Fras1 exhibited expression both in the UB as well as the comma-shaped bodies, and S-shaped bodies. Vwa2 expression was particularly pronounced in the superior aspect of the S-shaped body, between the distal tubule and the proximal tubule portion (Fig 3, indicated by arrowhead). Because of the interaction of Npnt with the FC (Fig 2), we then examined colocalization of Vwa2 with Npnt. Vwa2 and Npnt, demonstrated overlapping expression, however, to a much smaller extent than seen with Fras1 (predominantly UB) (S1 Fig).

Interface between the ureteric bud epithelium and metanephric mesenchyme of the developing kidney. All proteins in yellow are encoded by genes known to cause human CAKUT [1,37]. Red framed proteins (i.e. the according genes) have been reported to be causative of CAKUT in mice, if mutated [38–41]. All known causes of human or murine CAKUT depicted in this figure demonstrate a recessive pattern of inheritance.

VWA2, NPNT [42], FRAS1 and FREM2 are expressed by the ureteric bud epithelium, FRAS1 and FREM2 as transmembrane proteins [39,43]. GRIP1 (intracellular) is necessary for the correct positioning of FRAS1 and FREM2 at the basal side of the UB cell [43]. In a next step, FRAS1 and FREM2 are shed from the membrane and VWA2, NPNT, FRAS1 and FREM2 are translocated through the basement membrane. Together with FREM1 (produced in cells of the metanephric mesenchyme) FRAS1 and FREM2 form the Fraser-complex (FC) [40,44] with VWA2 as an accessory component [18]. The FC is essential for anchoring the BM to the underlying MM [40,44]. The FC hereby interacts with NPNT and indirectly with the integrin alpha 8 (α8)/integrin beta 1 (β1) dimer [44] and is thereby involved in GDNF signaling via the MM [38].

Reproduced with permission from van der Ven et al. *JASN* 29: 36–50, 2017.

### Secretion of monomeric Arg446Cys VWA2 is reduced

**Fig 4:**
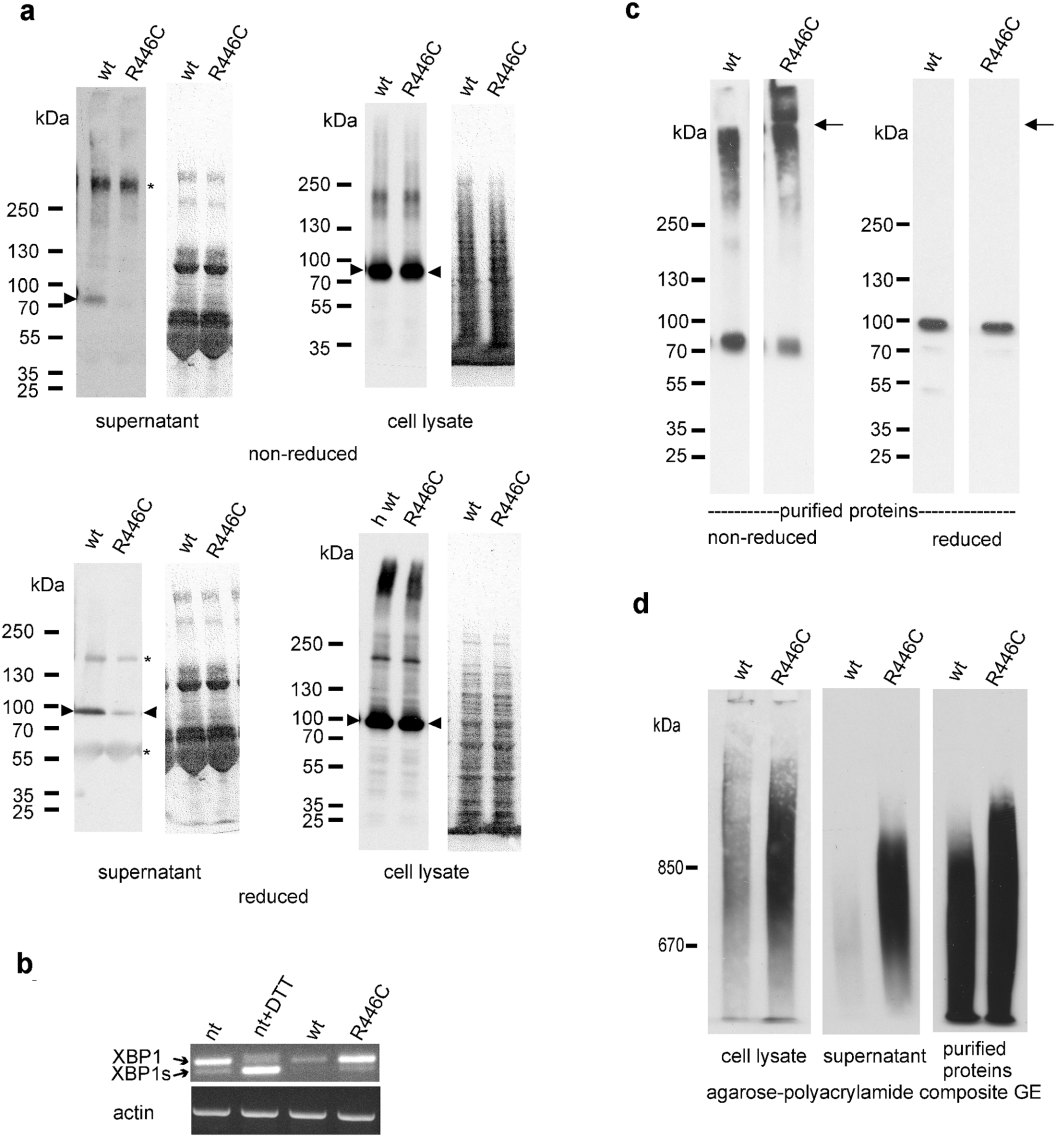
Reduced secretion and intracellular and extracellular aggregation of Arg446Cys VWA2. **(a)** Cell culture supernatants and cell lysates from wild type (wt) and Arg446Cys VWA2 (R446C) expressing 293EBNA cells were separated by SDS-PAGE under reducing and non-reducing conditions and detected with an antibody against the One-STrEP-tag. Arrowheads indicate monomeric VWA2. On the right, equal loading is demonstrated by Ponceau staining of the membranes. Asterisks indicate artefact bands. **(b)** cDNA from non transfected (nt), non-transfected ER stress induced (nt+DTT), wt VWA2 transfected (wt) and Arg446Cys VWA2 (R446C) transfected 293EBNA cells was submitted to RT-PCR and the PCR products separated by agarose gel electrophoresis. Arrows indicate the bands for XBP-1 and ER stress induced XBP-1s. Equal loading is demonstrated by actin control RT-PCR shown below. **(c)** Equal amounts (0.2 μg) of affinity purified wild type (wt) and Arg446Cys VWA2 (R446C) were separated by SDS-PAGE under reducing and non-reducing conditions and detected with an antibody against the C-terminal fragment (P3) of human VWA2. Under non-reducing conditions higher aggregates are seen. Arrows indicate the border between separation and stacking gel. (d) Equal amounts of cell culture supernatants and cell lysates from wild type (wt) and Arg446Cys VWA2 (R446C) expressing 293EBNA cells as in (a) and of affinity purified wild type (wt) and Arg446Cys VWA2 (R446C) as in (b) were separated by agarose-polyacrylamide composite gels under non-reducing conditions and detected with an antibody against the One-STrEP-tag.

To characterize the impact of the mutation on the function of VWA2, 293 EBNA cells were transfected with the mutated VWA2 construct in an expression vector that enables secretion of recombinant proteins. Equal amounts of full-length wild-type VWA2 or mutated VWA2 constructs were transfected into 293 EBNA cells and analyzed for differences in the ratio between protein levels in the supernatants and cell lysates after two days. In the supernatants, monomeric mutant VWA2 was strongly reduced compared to wild-type VWA2. This was the case under both reducing and non-reducing conditions (Fig 4a, column 1). The result indicates the formation of strongly disulfide-linked aggregates that do not enter normal SDS polyacrylamide gels. Indeed, agarose-polyacrylamide composite gel electrophoresis showed that mutated VWA2 is predominantly secreted as higher aggregates in contrast to wild type VWA2 (Fig 4d). Only a slightly increased amount of aggregates of mutated VWA2 was seen in cell lysates (Fig 4d). In contrast, cell lysates showed equal amounts of wild-type and mutant VWA2 monomers under both conditions (Fig 4a, column 2), suggesting that the Arg446Cys substitution leads to reduced secretion of monomeric VWA2 protein and increased secretion of disulfide-linked aggregated VWA2 protein. However, the slightly increased intracellular formation of aggregates of mutant VWA2 does not induce ER stress, because the spliced form of the X-box binding protein-1 mRNA (XBP1s) [45] was not elevated (Fig 4b).

### Arg446Cys VWA2 forms disulfide-linked aggregates

Since cysteine residues of secreted proteins—both in the secretory cellular compartments and in the extracellular space, can be subject to oxidation, leading to the formation of intra- and intermolecular disulfide bonds, we further investigated whether the Arg446Cys mutation might affect disulfide bond-dependent VWA2 aggregation. Due to the reduced secretion by freshly transfected 293EBNA cells (Fig 4a) monomeric Arg446Cys VWA2 could not be characterized further. However, after selection, transfected 293EBNA cells secreted enough mutant protein to enable purification from the cell culture supernatant via its N-terminal One-STrEP-tag. Equal amounts of wild-type and mutant VWA2 were analyzed on SDS polyacrylamide gels under reducing and non-reducing conditions (Fig 4b). Whereas under reducing conditions human wild-type and Arg446Cys VWA2 have the same mobility at about 90 kDa as the murine wild-type VWA2 monomer [17], they formed higher aggregates under non-reducing conditions that run at above 250 kDa. Strikingly, monomeric Arg446Cys mutant VWA2 was less abundant and aggregates of Arg446Cys mutant VWA2 were significantly larger and more abundant than those of the wild-type protein. Some of the mutant protein even did not enter the separating gel and remained in the stacking gel. Agarose- polyacrylamide composite gel electrophoresis confirmed that aggregate formation is more pronounced in purified Arg446Cys mutant VWA2 (Fig 4c). The aggregates run up to positions higher than 850 kDa. However, it should be noted that aggregation occurs also in purified wild type VWA2, as has been demonstrated by electron microscopy [17]. Together, this suggests that secreted mutant VWA2 protein forms less monomers and more and/or more stable intermolecular disulfide-bonds and thereby larger protein aggregates than its wild-type counterpartThis could mean mean that VWA2 cysteine residues are predominantly oxidized after their secretion, rather than already within the endoplasmatic reticulum and the Golgi apparatus.

## Discussion

We here discovered and characterized a homozygous missense mutation (Arg446Cys) in *VWA2* in a patient with high-grade bilateral VUR and consecutive development of ESRD. VWA2 has previously been demonstrated to interact with Fras1, a component of the Fraser complex (FC), *in vitro* [18]. Given the functional studies provided here, and given the established role of FC components and associated proteins in the pathogenesis of both syndromic as well as isolated CAKUT (Fig 2) [1,37,39,43,46], we conclude that the detected homozygous *VWA2* mutation very likely is causative for this patient’s CAKUT phenotype.

VWA2 is an extracellular matrix protein that was first identified and characterized by Sengle et al. [17]. It has previously been demonstrated to interact with the CSPG domain of Fras1 *in vitro* [18]. Fras1 is a component of the Fraser-complex. The FC is a ternary protein complex (FRAS1, FREM1, FREM2) that lines the extracellular epithelial-mesenchymal interface (Fig 2) [40]. As such, the FC has been shown to play a role in the crosstalk between the epithelial component (the ureteric bud, [UB]) and the mesenchymal component (the metanephric mesenchyme, [MM]) of the developing kidney during the processes of renal morphogenesis [46]. Loss of function of any of the three components disrupts the FC and leads to the phenotype of Fraser syndrome (FS) [39,40,46]. FS is a severe syndromic phenotype characterized by manifestations including cryptophthalmos, syndactyly as well as CAKUT [39,46].

Our group previously demonstrated that missense variants in genes encoding members of the FC as well as the FC interactor GRIP1 cause isolated CAKUT phenotypes in humans as opposed to the syndromic FS-phenotype that occurs due to abrogating mutations in these genes [1]. Recessive mutations in genes that encode direct and indirect interactors of the FC such as *nephronectin (Npnt)* and *integrin alpha 8 (ITGA8)* have independently been demonstrated to be causative of isolated CAKUT phenotypes in mice (*Npnt*) and humans (*ITGA8*), respectively, thus further indicating the relevance of the FC and associated proteins for the development of isolated CAKUT [37,38,44] (Fig 2).

Like Fras1, Vwa2 is highly expressed in tissues of the developing kidney of the mouse [17,47] (Fig 3, S1 Fig). However, Vwa2 is not detectable in the kidney of adult mice [17], thereby indicating its potential importance for the embryonic development of the kidneys and the urinary tract.

By applying immunohistochemistry, we demonstrate that Fras1 and Vwa2 co-localize in the nephrogenic zone in the kidney of newborn (P1) mice (Fig 3). The nephrogenic zone constitutes an area in the renal cortex of newborn mice where postnatal nephron formation continues for approximately 3 days. Coronal sections of the nephrogenic zone have the highest informative value in regards to protein expression in developing structures of the kidney (UB, derivatives of the MM) and consequently allow for an assessment of a protein’s relevance for the pathogenesis of CAKUT.

We found a pronounced localization of Vwa2 in the basement membrane of the UB and derivatives of the MM (in comma-shaped bodies and S-shaped bodies). This distribution is compatible with a role of Vwa2 in the pathogenesis of CAKUT phenotypes.

We also performed a series of experiments to elucidate how the p.Arg446Cys amino acid exchange in the mutant VWA2 might change the biochemical properties and thereby the function of the protein. Strikingly, in our cell culture system we observed strongly reduced amounts of monomeric mutant VWA2 protein in the supernatant and increased secretion of disulfide-linked aggregated protein, while total intracellular levels were unaltered, pointing to reduced secretion and, possibly, a loss-of-function effect of the mutation. However, previous experiments indicated no overt phenotype caused by antisense-mediated inactivation of *vwa2* in zebrafish [18], suggesting that the Arg446Cys missense mutation in our CAKUT patient has a gain- rather than a loss-of-function effect. Yet, while most gain-of-function mutations are dominantly inherited, the recessive inheritance of the Arg446Cys missense mutation points to a strong dose-dependence of the altered VWA2 protein function. Furthermore, among gain-of-function effects, geneticists distinguish between hypermorphic effects with an increased by otherwise normal activity, and neomorphic effects with a novel but harmful activity of the mutated protein. Although further experiments will be required to provide ultimate proof for this notion, our data point to a neomorphic effect of the Arg446Cys exchange in VWA2, triggered by the creation of an unpaired cysteine residue, which could have consequences inside and outside of the VWA2 producing cells. This could be due to the increased formation of higher aggregates.

Mature wild type VWA2 contains an even number of 26 cysteine residues and it is likely that six of these form disulfide bonds that connect the N- and the C-terminus in every single VWA domain, 12 are involved in the formation of disulfide bonds within the two EGF domains and six most likely pair within the cysteine rich domain. The status of the two remaining cysteine residues in the first and the third VWA domain remains unclear, but they may form an intramolecular disulfide bond between both VWA domains. It is furthermore possible that disulfide shuffling of these two residues is responsible for the formation of aggregates seen for recombinantly expressed VWA2 (Fig 4b and 4c and [17]). However, the Arg446Cys mutation in the second VWA domain causes an odd number of cysteine residues which could lead to severe folding problems within the secretory pathway or outside of the cell. Indeed, the introduction of a cysteine residue led to a reduced secretion of the mutant protein which could be due to a change in the fold of the remaining VWA, EGF or cysteine rich domains or to an intermolecular disulfide bond to other proteins present in the secretory pathway. For example, a disease-causing Arg to Cys mutation which induced misfolding of human cationic trypsinogen is found in a form of hereditary pancreatitis. The endoplasmic reticulum (ER) stress markers immunoglobulin-binding protein (BiP) and the spliced form of the X-box binding protein-1 (XBP1s) were elevated in cells expressing the mutant trypsinogen [48]. However, we could not detect elevated levels of XBP1s in EBNA293 cells expressing Arg446Cys VWA2 indicating that the mutation does not induce ER stress. The free cysteine residue may also form disulfide bonds with other proteins in the secretory compartments and affect their folding and secretion. On the other hand we could clearly demonstrate that extracellular Arg446Cys mutant protein forms disulfide-linked higher aggregates, which could lead to a misassembled Fraser complex. A larger cross-linked complex could affect its interplay with basement membranes and cellular receptors.

## Conclusion

We have discovered and characterized a homozygous missense mutation (Arg446Cys) in *VWA2* in a patient with high-grade VUR and consecutive development of ESRD. We hereby identified another Fraser-complex-related, extracellular matrix protein, VWA2 (Fig 2) that likely causes isolated congenital anomalies of the kidney and urinary tract. Vwa2 interacts with Fras1 of the FC, and thereby constitutes a promising candidate gene for causing disease in additional individuals with CAKUT. The fact that no further recessive missense mutations of VWA2 were detected when examining another ~1,000 individuals with CAKUT is not surprising, given that the Arg466Cys mutation most likely represents a specific dose-dependent neomorphic mutation that affects intra- and/or intermolecular disulfide bond formation, thereby creating a protein with novel and harmful biochemical properties and functions. Such recessive gain-of-function (neomorphic) mutations are known to be particularly rare as they require the recessive exchange of a very specific amino acid residue to a very specific altered residue.

By applying immunohistochemistry, we demonstrate that Fras1 and Vwa2 co-localize in the nephrogenic zone of newborn mice and hereby exhibit a distribution pattern that is highly suggestive of a role in the development of the kidney and the urinary tract. Our experiments *in vitro* indicated a potential dose-dependent, neomorphic effect of the Arg446Cys missense mutation.

## Supporting information

S1 Fig Vwa2 co-localizes with Npnt in the nephrogenic zone of newborn mice. Immunohistochemistry on coronal sections of the nephrogenic zone of newborn (P1) mice. Figures display representative staining results obtained from kidney sections of two different animals. Experiments were performed independently and yielded similar results. Staining for Vwa2 (green) and nephronectin (Npnt, red) reveals a partial co-localization, predominantly in the ureteric bud (UB) as seen by the yellow-appearing structures in the “merge” panel on the very right. The two proteins, however demonstrate overlapping expression to a much smaller extent than Vwa2 and Fras1 (Fig 3). Vwa2 (green) can be also identified without concurrent expression of Npnt (red), and *vice versa*. SS; S-shaped body.
(TIFF)

S1 Table Filtering process for variants from hg19 reference sequence following WES in individual A3964-21 from consanguineous descent and isolated CAKUT. (TIFF)

## Abbreviations

CAKUT: Congenital anomalies of the kidney and urinary tract
CKD: Chronic kidney disease
CNV: Copy number variation
ESRD: End-stage renal disease
FC: Fraser complex
FS: Fraser syndrome
HM: Homozygosity mapping
MM: Metanephric mesenchyme
UB: Ureteric bud
VUR: Vesicoureteral reflux
WES: Whole exome sequencing
