# Supplementary figures and images for: A homozygous missense variant in VWA2, encoding an interactor of the Fraser-complex, in a patient with vesicoureteral reflux
Source: PLoS One. 2018 Jan 19;13(1):e0191224. doi: 10.1371/journal.pone.0191224 (PMC5774751; doi:10.1371/journal.pone.0191224)

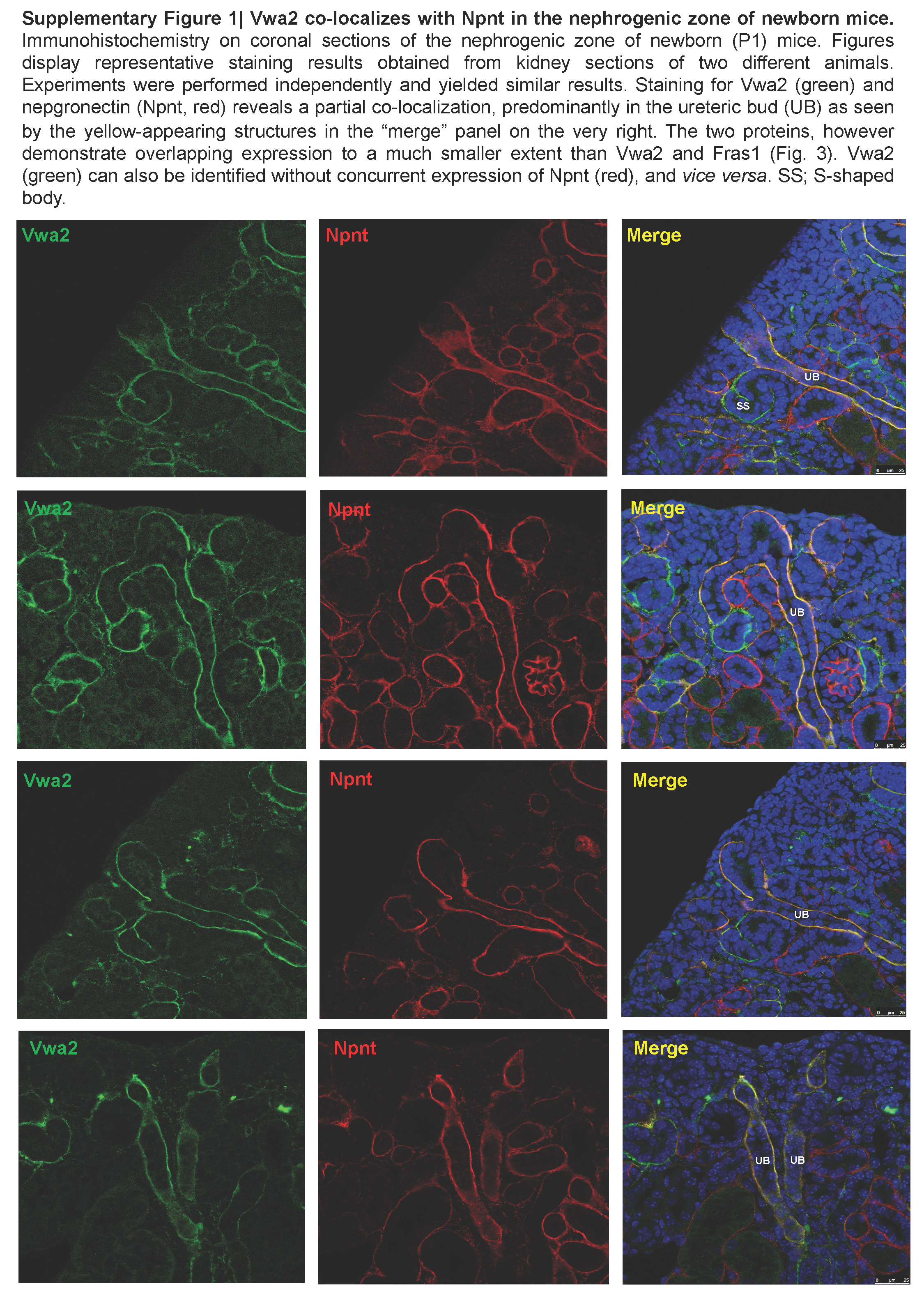

Supplement: S1 Fig — Immunohistochemistry on coronal sections of the nephrogenic zone of newborn (P1) mice. Figures display representative staining results obtained from kidney sections of two different animals. Experiments were performed independently and yielded similar results. Staining for Vwa2 (green) and nephronectin (Npnt, red) reveals a partial co-localization, predominantly in the ureteric bud (UB) as seen by the yellow-appearing structures in the “merge” panel on the very right. The two proteins, however demonstrate overlapping expression to a much smaller extent than Vwa2 and Fras1 (Fig 3). Vwa2 (green) can be also identified without concurrent expression of Npnt (red), and vice versa. SS; S-shaped body. (TIFF) [file pone.0191224.s001.tiff]

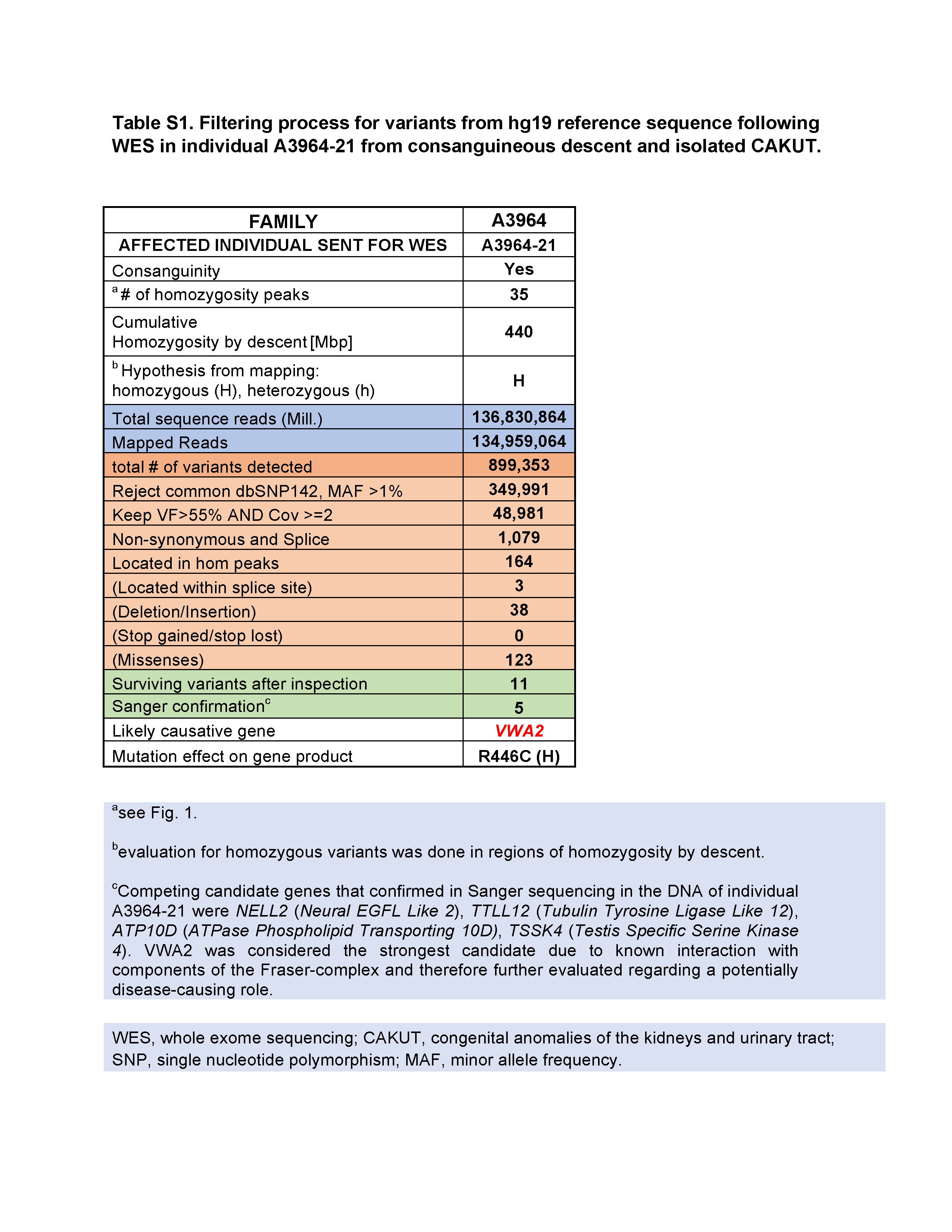

Supplement: S1 Table — (TIFF) [file pone.0191224.s002.tiff]
